# Longitudinal Outcomes and Monitoring of Patients With Multisystem Inflammatory Syndrome in Children

**DOI:** 10.3389/fped.2022.820229

**Published:** 2022-04-01

**Authors:** Michael A. Fremed, Kanwal M. Farooqi

**Affiliations:** Division of Pediatric Cardiology, Department of Pediatrics, Columbia University Vagelos College of Physicians and Surgeons, NewYork-Presbyterian-Morgan Stanley Children's Hospital, New York, NY, United States

**Keywords:** longitudinal outcomes, multisystem inflammatory syndrome in children, COVID-19, myocarditis, pandemic

## Abstract

The acute manifestations and short-term outcomes of multisystem inflammatory syndrome (MIS-C) have been extensively described; however, our understanding of the longitudinal outcomes associated with this condition continue to evolve. Here we review the existing literature on outcomes of MIS-C up to 1 year following diagnosis and summarize current published expert recommendations for management and follow up of these patients.

## Introduction

The multisystem inflammatory syndrome in children (MIS-C) is now a well described phenomenon associated with novel coronavirus 2019 disease (COVID-19) (1–9). It primarily impacts children following SARS-CoV-2 infection, though a similar phenomenon has more recently been described in adults (10). Cardiac injury in addition to gastrointestinal manifestations is a predominant feature of this condition, with up to 80% of MIS-C cases involving cardiac insult (2, 4, 11). Common gastrointestinal symptoms include abdominal pain, emesis, and diarrhea. Additionally, many of these patients require admission to the intensive care unit as well as vasoactive support at rates more than 5 times that of patients with severe acute COVID-19 (12).

More recently, the focus has shifted toward follow up of these patients and long-term complications associated with MIS-C, though the existing data on outcomes post-discharge in these patients is limited. Here, we summarize the current literature on post-discharge outcomes in children with MIS-C and use this evidence to provide expert opinion-based recommendations for how to monitor and manage these patients in both acute and post-acute time periods.

## Acute MIS-C

The acute presentation of MIS-C is defined by the Centers for Disease control as fever, laboratory evidence of inflammation, hospitalization for clinically severe illness in individuals under 21 years of age with positive SARS-CoV-2 testing (PCR, serology, or antigen) with two or more organ systems involved for which there is no plausible alternative diagnosis (13). Cardiac injury has consistently been shown to be a key feature of this condition. The findings from the largest cohorts of MIS-C patients are summarized in Table 1. In one of the larger multicenter studies characterizing this disease, Feldstein et al. (2) reported on 216 children with MIS-C. In this cohort, 92% of patients had gastrointestinal manifestations and 80% demonstrated cardiac involvement. Hematologic abnormalities were present in 76% of patients, mucocutaneous findings were present in 74% of patients, and respiratory system was involved in 70% of patients. Specific cardiac manifestations of MIS-C include elevated troponin and NT-proBNP (2, 3, 11, 14) ventricular dysfunction, myocarditis/pericarditis, arrhythmia, ECG abnormalities including first degree atrioventricular block (15), prolonged QTc, T wave abnormalities, and ST segment changes, and pericardial effusions, among others (2, 3, 11, 15–17).

**Table 1:** Acute Outcomes in Patients with MIS-C.

	^*^Feldstein et al. (*n =* 539)	**Haslak et al. (*n =* 76)**	**Valverde et al. (*n =* 286)**	**Ahmed et al. (*n =* 662)**
**Demographics**
Age (years)	8.9	8.2 (±4.4)	8.4 (IQR 3.8-12.4)	9.3 ± 0.5
Male	58	55	68	52
Underlying comorbidity	31	16	6	48
**Organ system involved**
Any cardiovascular	67	93	NA	54
Mitral valve regurgitation	NA	47	42	3
Tricuspid valve Regurgitation	NA	NA	6	NA
Systolic dysfunction	34	18	38	45
Coronary artery dilation/aneurysm	13	8	24	16
Pericardial Effusion/Pericarditis	25	17	21	22
Gastrointestinal	90	50	71	74
Dermatologic	67	53	63	56
Respiratory	80	45	56	NA
**Admission characteristics**
ICU	74	33	57	71
Vasoactives/inotropes	NA	29	NA	52
Hospital LOS (days)	7 (IQR 5–11)	8 (Range 2–22)	10 (IQR 3–7)	7.9 ± 0.6
**Respiratory support**	56	30	NA	40
Supplemental oxygen only	NA	14	NA	NA
Non-invasive ventilation	36	12	NA	7
Invasive mechanical ventilation	18	4	15	22
ECMO	3	NA	≤ 1%	4
Death	2	1	≤ 1%	2
**Treatment**
IVIG	77	100	78	76
Steroids	70	97	28	52
Anticoagulation	63	92	38	26
Anakinra	NA	4	NA	9
Plasmapheresis	NA	18	NA	NA
Antibiotics	NA	NA	71	16
Antiplatelet	57	NA	74	17

*Numbers represent % of sample unless otherwise stated. Continuous variables are reported as median (IQR or Range) or mean ±standard deviation. Categorical variables are reported as percent of study sample*.

*ECMO, Extracorporeal membrane oxygenation; IVIG, Intravenous immune globulin*.

* *Feldstein et al. (n = 539)- only percentages of severe outcomes are reported in this study*.

Studies of echocardiographic findings have shown ventricular systolic dysfunction in ~40–60% of patients with acute MIS-C (2, 11, 12, 18, 19) as well as evidence of diastolic dysfunction, even in the absence of systolic dysfunction. Coronary artery abnormalities such as aneurysm and dilation have been demonstrated but are less common than in classic Kawasaki disease (20). Left ventricular global longitudinal strain may be abnormal even in the absence of clinically apparent cardiac symptoms and left ventricular systolic dysfunction (21). Abnormal strain has been shown to be associated with increased odds of vasoactive support requirement, duration of vasoactive support, and ICU length of stay (22). An association has also been shown between degree of inflammation and cardiac injury severity, in particular CRP and NT-proBNP (11). Abnormal strain has also been associated with greater number of organ systems involved (23).

In a 2020 systematic review, Ahmed et al. (24) reported on 39 observational studies of MIS-C patients encompassing the period January 1st 2020 to July 25th 2020. Out of 662 patients, 71% required admission to the intensive care unit (ICU). Mechanical ventilation was utilized in 22% of these patients and 4% required extracorporeal membrane oxygenation (ECMO). Notably, nearly half of these patients had underlying comorbidities, with obesity present in 50% of those with comorbidities. Plasmapheresis has also been utilized as a treatment option for MIS-C (25), with one small prospective trial showing a significant decrease in organ dysfunction in those who underwent plasmapheresis in addition to steroids and intravenous immune globulin (IVIG) as compared to those who received steroids and IVIG only (26).

Gastrointestinal manifestations are common in MIS-C and are part of the initial presentation in up to 90% of cases. Symptoms can range from more mild nausea, vomiting, or diarrhea to more severe involvement, such as appendicitis, pancreatitis, hepatitis or hepatomegaly, intussusception, biliary sludge or cholecystitis, ascites, ileitis or colitis, abdominal fluid collections, and mesenteric adenitis (12, 25, 27, 28). Valverde et al. (4) looked at 286 patients with MIS-C, 159 of whom underwent abdominal ultrasound. Of those who underwent ultrasound, 39% had abnormal abdominal findings including ascites in 21%, lymphadenopathy in 14%, ileitis in 9%, and colitis in 4%.

The immunological profile of this disease is similar to that of Kawasaki disease with some notable exceptions. Lymphocytopenia and thrombocytopenia suggestive of bone marrow suppression are common in the acute phase of MIS-C, as opposed to Kawasaki disease, where thrombocytosis is more common than thrombocytopenia and CRP may not be as elevated (29). Syrimi et al. (30) performed deep immune profiling of the various stages of MIS-C and showed that in addition to the neutrophilia there is higher activation of monocytes, memory CD8+ cells, T-cells, B-cell plasmablasts, and double negative B-cells. Additionally, they found high IL-6 and systemic complement activation with high C5b-9 levels. Because MIS-C is believed to be an immune-mediated phenomenon that triggers a hyper-inflammatory response, management has largely relied on antagonists of various components of the immune system (31). Treatment of MIS-C has typically involved steroids, IVIG, and/or anakinra, an IL-1 inhibitor (32). Aspirin is also commonly used, in particular when there is concern for coronary artery involvement, similar to treatment for Kawasaki disease (33).

Despite the illness severity in these patients, mortality is low with reported rates around 2% (24) with 1 case report of sudden cardiac death occurring in a patient after initial clinical improvement after MIS-C (34). In a large multicenter study of 112 COVID-19 related deaths in children under age 21, McCormick et al. found that only 14% of deceased patients met criteria for MIS-C. Mortality after MIS-C was more likely to occur in previously healthy patients while mortality due to non-MIS-C COVID-19 was more likely in those with underlying comorbidities (35).

Several prognostic indicators have been suggested based on the available evidence, In an outcomes study of 76 patients with MIS-C, Haslak et al. showed that those admitted to the pediatric ICU were generally older in age and had lower albumin on admission. Higher procalcitonin was also associated with longer hospital length of stay (25). Similar findings were reported by Abrams et al. in a large, retrospective surveillance study, in which they reviewed 1,080 cases of MIS-C. They found that the odds of ICU admission were greatest in those 13–20 years old and non-Hispanic Black patients. Shortness of breath, abdominal pain on presentation, decreased ventricular function, shock, or myocarditis were all associated with higher rates of ICU admission (36). Recent reports from the CDC and Levy et al. suggest a significant protective effect of mRNA based COVID-19 vaccination in children 12 years and older against development of MIS-C, especially in severe cases (37, 38).

## Longitudinal Outcomes Following MIS-C (Table 2)

### Immunological

Rapid normalization of both inflammatory markers and cardiac injury biomarkers has been demonstrated following the acute phase of the illness. Laboratory abnormalities including NT-proBNP, troponin-T, lymphocytopenia, thrombocytopenia, and C-reactive protein (CRP) appear to return to normal ranges by 1–4 weeks post discharge (11). In a study of 1 year outcomes of UK patients with MIS-C, Davies et al. reported that 50% of patients demonstrated normalization of lymphocytes and neutrophils within a few days post-admission, CRP within 11 days, and ferritin and D-dimer within 3 weeks. At 1 year post-admission, only 2% of patients had still not demonstrated normalization of lymphocytes, neutrophils, and platelet count. D-dimer remained abnormal in 25% of patients and Ferritin remained abnormal in 17% of patients at 1 year (39).

**Table 2:** Longitudinal Outcomes MIS-C.

	**Farooqi et al.** **(*n =* 45)**	**Capone et al.** **(*n =* 50)**	**Barris et al.** **(*n =* 16)**	**Penner et al.** **(*n =* 46)**	**Matsubara et al.** **(*n =* 60)**
Follow up (months)	5.8 (IQR 1.3–6.7)	6–8	7 (IQR 6.0–8.4)	6	3–6
**Cardiovascular**
**Any echo abnormality (at least mild)**
Admission	80	66	NA	33	NA
Short Term	18	21	NA	NA	NA
Mid Term	8	4	NA	4	
**Tricuspid Valve regurgitation**	27			NA	NA
Admission	60		NA		
Short Term	15		NA		
Mid Term	4		NA		
**Mitral valve regurgitation**
Admission	36		NA	NA	NA
Short Term	78		NA		
Mid Term	4		NA		
**Left ventricular systolic dysfunction**
Admission	49	52	44	NA	NA
Short term	10	2	13		NA
Mid term	4	0	0	0	NA
**Coronary artery dilation or aneurysm**
Admission	16	24	19		7
Short term	0	2	6	4	2
Mid term	0	0	6	4	0
**Pericardial effusion**
Admission	44	NA	NA	2	
Short term	10	NA	NA	2	
Mid term	0	NA	NA	2	
**CMR performed**	NA	22	56	NA	23
Timing of MRI (post-admission)	NA	2–4 weeks	9.4 (Range: 8.8–10.0) months	NA	5 days−9.5 months
Myocardial edema	NA	0	44	NA	21
Fibrosis	NA	0	0	NA	14
**6-min walk exercise test (abnormal)**
6 weeks	NA	NA	NA	65	NA
6 month	NA	NA	NA	45	NA
**Gastrointestinal**
Symptoms					
Admission	NA	NA	NA	98	NA
Mid term	NA	NA	NA	13	NA
Significant imaging findings	NA	NA	NA	33	NA
**Labs (weeks to normalization post-discharge)**
Troponin	1–4	2	NA	6	NA
BNP	1–4	2	NA	6	NA
Platelets	0	8	NA	6	NA
CRP	1–4	2	NA	6	NA

*NA, not available*.

*Numbers represent % of sample unless otherwise stated. Continuous variables are reported as median (IQR or Range) or mean ± standard deviation. Categorical variables are reported as percent of study sample*.

### Cardiovascular

In one of the first studies to report on long term follow up following MIS-C, Farooqi et al. (11) followed 45 patients up to 9 months following diagnosis. Despite an 80% prevalence of at least mild and 44% prevalence of at least moderate-severe echocardiographic abnormalities, only 18% had residual mild abnormalities by 1–4 week follow up. NT-proBNP and Troponin-T normalized in most patients by 1–4 weeks. Of the 24 patients who returned for 4–9 month follow up, only 2 patients had remaining echocardiographic abnormalities: 1 with mild ventricular dysfunction and 1 with mild atrioventricular valve regurgitation. Kobayashi et al. (23) showed that left ventricular strain may remain abnormal even after normalization of left ventricular ejection fraction; however, a mid-term longitudinal study by Matsubara et al. (40) demonstrated that several strain paramaters rapidly improve in the first week following presentation then continue to improve more gradually through 3–4 months. In their study, abnormalities in multiple strain parameters were worse in those with biochemical evidence of cardiac injury such as elevated troponin or BNP; however, they also improved more rapidly and normalized within the same time period as those without biochemical evidence of cardiac injury.

Capone et al. (18) conducted a similar study following 50 MIS-C patients over a 6-month period at intervals of 2, 8 weeks, and 6 months post-discharge. During the acute phase, 52% of subjects demonstrated evidence of left ventricular systolic dysfunction on echocardiogram and 32% showed evidence of left ventricular diastolic dysfunction, and 24% had coronary artery aneurysm or dilation. By 2 weeks, left ventricular systolic dysfunction was present in only 1 patient (2%) and coronary artery abnormalities were present in 3 patients (6%) with complete resolution by 8 weeks. Despite these rapid improvements, diastolic dysfunction persisted in 11% at 2 weeks, 9% at 8 weeks, and 4% at 6 months. Penner et al. (14) found that nearly all cardiac abnormalities including serum biomarkers and ventricular dysfunction normalized by 6 weeks post-diagnosis, with only 1 patient demonstrating persistent large coronary artery aneurysm at 6 weeks, with no change in size at 6 months, and 1 pericardial effusion. An additional patient demonstrated persistent but improved coronary artery dilation at 6 months.

Davies et al. (39) reported on a cohort of MIS-C patients 1 year after their initial admission to the ICU and found that ventricular dysfunction resolved by day 74 post-admission. Out of 19 coronary artery aneurysms, 14 had resolved at 1 year follow up, as did 9/10 “bright” coronary arteries. The remaining patient with bright coronary arteries progressed to an aneurysm. There was no evidence of ventricular dysfunction in any patient after 50 days post-admission and 75% of patients had normalization of function by day 21 post-admission.

Because of the severity of cardiac injury seen during the acute phase of MIS-C, cardiac MRI (CMR) has become a useful imaging modality in this population. Despite the myocarditis like presentation, MIS-C differs from classic viral myocarditis in that it appears to be predominantly, if not purely, a post-infectious inflammatory mediated process. Several studies have looked at cardiac MRI findings in patients following hospitalization for MIS-C. Some of these have found findings consistent with myocardial edema. However, most have not shown evidence of the necrosis, fibrosis, or late gadolinium enhancement that is typically seen in cases of classic viral myocarditis on studies performed 8 days to 3 months post-diagnosis (18, 41–44). It is possible that when fibrosis does occur it is more common in the first 2 weeks following diagnosis as compared to later periods, as demonstrated in a study by Matsubara et al. In this report, 2/14 (14%) CMR showed evidence of fibrosis (as well as edema), performed at 6 and 10 days post-diagnosis, respectively. Both of these patients had no evidence of left ventricular dysfunction by conventional echocardiography, strain, or CMR. Although CMR appears to be normal in most cases following resolution of acute illness, one small case series of CMR findings in patients with MIS-C did show late gadolinium enhancement in two patients at 50 days and 3.5 months following diagnosis (45). Similarly, in a small prospective controlled study of CMR findings in children following COVID-19 or MIS-C, Webster et. al. found no evidence of myocardial injury in CMR findings compared with healthy controls (46). Similar CMR findings are described in adults with multisystem inflammatory syndrome, with myocardial edema being the predominant finding and fibrosis or necrosis a more rare abnormality (47). These findings suggest that the cardiac injury associated with MIS-C is a result of a transient hyperinflammatory process and that long term abnormalities and cardiac injury is likely rare.

### Gastrointestinal

Penner et al. (14) reported outcomes from 46 patients in the UK up to 6 months following MIS-C. They found that gastrointestinal symptoms including abdominal pain, diarrhea, nausea and vomiting resolved by this time point. Fecal calprotectin was elevated in 7% of patients for whom this data was available. On abdominal imaging, 20% of patients had abnormalities at 6 weeks including ileitis, colitis, liver inflammation, and splenomegaly, with 1 patient experiencing persistent splenomegaly at 6 months post-diagnosis. One patient underwent colonoscopy and gastroscopy and demonstrated eosinophilic inflammatory changes in the colon and ileum. Liver enzymes continued to rise through 6 weeks before normalizing. Additionally, the median BMI continued to increase through the 6 month follow up (Table 2).

### Quality of Life

In the same 1 year follow up study, Penner et al. performed quality of life assessments using the validated PedsQL (48) assessment. They found severe impairment in physical functioning in up to 13% of participants. Emotional lability was present in 26% at 6 weeks and 15% at 6 months. Assessment with the Pediatric Index of Emotional Distress (49) showed 7% of patients scored above the cutoff for high risk of significant distress. Parental anxiety regarding possibility of MIS-C relapse was high (31%). Despite these concerns, 73% of parents indicated some degree of hesitancy with regards to COVID-19 vaccination. Nearly all children were back in school by 6 months post-diagnosis (14).

## Return to Physical Activity Following MIS-C

Given the high incidence of cardiac injury including myocardial dysfunction associated with MIS-C, a major concern following resolution of the acute illness has been returning to physical activity including both organized competition as well as routine daily activities. Current recommendations have adopted the clinical recommendations for traditional myocarditis to guide management in these patients, which includes exercise restriction for 3–6 months (50–52); however, given the growing MRI evidence that this is not the same as the typical myocarditis, this may warrant additional consideration in the future. Even in the absence of clinical evidence of myocardial injury, deconditioning has been demonstrated in healthy children likely due to increased sedentary behavior during periods of quarantine and lockdowns, with studies showing decreased performance on cardiopulmonary exercise testing and 6- min walk testing. In their 1 year follow up study, Penner et al. found that 65% of MIS-C patients were below the 3rd percentile for distance at 6 weeks and 45% of patients were below the 3rd percentile for distance at 6 months. Regardless, all patients with MIS-C should undergo cardiology consultation prior to discharge (if admitted) with regular outpatient cardiology follow up post discharge and should be cleared by a cardiologist prior to returning to physical activity. Once cleared, they should undertake a gradual approach over a minimum of a 7 day period before returning to full intensity and duration (52–54).

## Current Recommendations

The American College of Rheumatology published guidance on the management of patients with MIS-C (32) and more recently submitted an updated version for publication, available on their website (55). In this report they recommend a basic lab workup for all children under investigation for MIS-C including complete blood count, complete metabolic panel, ESR, CRP, and SARS-CoV-2 testing (PCR and/or serology). If the results of this initial screening round of testing are suspicious for MIS-C or the patient experienced shock of unclear etiology, additional testing should be obtained including additional acute phase reactants, cardiac enzymes such as BNP and troponin T, SARS-CoV-2 serology if not already obtained, peripheral blood smear, ECG, and echocardiogram. If a diagnosis of MIS-C is made, patients with mild symptoms may be appropriate to manage with close monitoring only. For those requiring hospitalization, first line therapy should include both IVIG 2 gm/kg and steroid therapy. If fevers and/or end-organ damage persists despite initial treatment, intensification treatment should be provided with high dose steroids, anakinra, or infliximab. As of the time the guidance was published, they did not recommend tocilizumab or other immunomodulatory agents due to insufficient evidence unless glucocorticoids and anti-IL1 therapies are contraindicated or the illness is refractory to these therapies.

For those patients with abnormal cardiac biomarkers on admission, BNP and/or troponin T should be trended until normalized. ECGs should be performed at least every 48 h during admission and followed up post-discharge, with continuous telemetry in the event of abnormal ECG findings as well as home ambulatory monitoring. Echocardiography should be performed at diagnosis, as clinically indicated during admission, and repeated at 1–2 and 4–6 weeks following presentation with possible 1 year follow up. A cardiac MRI should be considered 2–6 months post-diagnosis for those patients with significant left ventricular dysfunction. Cardiac CT should be performed on patients for whom there is suspicion of coronary artery abnormalities but are not able to be visualized adequately by echocardiography.

With regards to thromboprophylaxis, the ACR recommends low dose-aspirin for all patients with MIS-C until normalization of the platelet count. Patients with coronary artery aneurysms with Z scores >10 should also receive enoxaparin or warfarin. Those at higher risk for thromboembolic events including patients with central venous lines, age >12 years, malignancy, admitted to the ICU, severely depressed left ventricular dysfunction, and markedly elevated D-dimer values should also be considered for higher intensity anticoagulation.

At our institution, we have a dedicated MIS-C follow up team consisting of pediatric cardiologists with follow up visits conducted at 2, 6 weeks, 6 months, and 1 year following diagnosis. Bloodwork, ECG, and echocardiography are performed at each visit and patients are exercise restricted for 3–6 months. CMR is performed in the acute phase of MIS-C if mild or more significant left ventricular dysfunction is present (ejection fraction ≤50%) and in all patients at 6 months following diagnosis. Longitudinal outcomes studies are ongoing with patients expected to be followed through at least 1-year post-discharge and potentially longer. Large, federally funded multi-center trials are also underway including the NIH sponsored Long-terM OUtcomes after the Multisystem Inflammatory Syndrome in Children (MUSIC) study, which aims to follow at least 600 patients across North America for 2 years following initial MIS-C presentation (56). The MIS-C population will also be included in the NIH RECOVER program, a large scale research initiative designed to study post-acute sequelae of COVID-19, which includes MIS-C and “Long-COVID” (57).

## Conclusions

MIS-C is a severe consequence of COVID-19 in children that is associated with significant hemodynamic and cardiovascular compromise. The acute presentation of MIS-C typically includes hypotension, fever, elevated inflammatory markers, gastrointestinal and cardiac manifestationsthat require management in the intensive care unit. Despite the severity of initial presentation, most abnormalities appear to quickly resolve within the first few weeks. In addition,despite clinically presenting with a myocarditis-like picture, findings on CMR that are consistent with myocarditis such as myocardial fibrosis are rare. Though we continue to expand our knowledge of this condition, providers should continue to take a cautious approach to the management of this patient population until the long-term outcomes following MIS-C are more completely understood. The results from various federally funded research initiatives studying long-term outcomes in this patient population will be crucial to our understanding of this disease and will allow for more definitive clinical guidelines for management of these patients.

## Author Contributions

MF and KF contributed to conception and design of the study and contributed meaningfully to the writing and revision of the manuscript in its submitted form. All authors contributed to the article and approved the submitted version.

## Funding

This publication was supported in part by Genentech ML42866 and by 3U01AI100119-09S1.

## Conflict of Interest

The authors declare that the research was conducted in the absence of any commercial or financial relationships that could be construed as a potential conflict of interest.

## Publisher's Note

All claims expressed in this article are solely those of the authors and do not necessarily represent those of their affiliated organizations, or those of the publisher, the editors and the reviewers. Any product that may be evaluated in this article, or claim that may be made by its manufacturer, is not guaranteed or endorsed by the publisher.

## References

[1] Kim L Whitaker M O'Halloran A Kambhampati A Chai SJ Reingold A. COVID-NET Surveillance Team. Hospitalization Rates and Characteristics of Children Aged <18 Years Hospitalized with Laboratory-Confirmed COVID-19 - COVID-NET, 14 States, March 1-July 25, 2020. MMWR Morb Mortal Wkly Rep. (2020) 69:1081–8. 10.15585/mmwr.mm6932e3 32790664 PMC7440125

[2] Feldstein LR Rose EB Horwitz SM Collins JP Newhams MM Son MBF. Overcoming COVID-19 Investigators) CDC COVID-19 Response Team. Multisystem inflammatory syndrome in us children and adolescents. N Engl J Med. (2020) 383:334–46. 10.1056/NEJMoa2021680 32598831 PMC7346765

[3] Cheung EW Zachariah P Gorelik M Boneparth A Kernie SG Orange JS. Multisystem Inflammatory Syndrome Related to COVID-19 in Previously Healthy Children and Adolescents in New York City. JAMA. (2020) 324:294–6. 10.1001/jama.2020.10374 32511676 PMC7281352

[4] Valverde I Singh Y. Sanchez-de-Toledo J Theocharis P Chikermane A Di Filippo S. Acute cardiovascular manifestations in 286 children with multisystem inflammatory syndrome associated with COVID-19 infection in Europe. Circulation. (2021) 143:21–32. 10.2139/ssrn.3634853 33166189

[5] Zachariah P Johnson CL Halabi KC Ahn D Sen AI Fischer A. Columbia Pediatric COVID-19 Management Group. Epidemiology, Clinical Features, and Disease Severity in Patients With Coronavirus Disease 2019 (COVID-19) in a Children's Hospital in New York City, New York. JAMA Pediatr. (2020) 174:e202430. 10.1001/jamapediatrics.2020.2430 32492092 PMC7270880

[6] Spencer R Closson RC Gorelik M Boneparth AD Hough RF Acker KP. COVID-19 inflammatory syndrome with clinical features resembling Kawasaki Disease. Pediatrics. (2020) 146:e20201845. 10.1542/peds.2020-1845 32843441

[7] Verdoni L Mazza A Gervasoni A Martelli L Ruggeri M Ciuffreda M. An outbreak of severe Kawasaki-like disease at the Italian epicentre of the SARS-CoV-2 epidemic: an observational cohort study. Lancet. (2020) 395:1771–8. 10.1016/S0140-6736(20)31103-X 32410760 PMC7220177

[8] Cattalini M Della Paolera S Zunica F Bracaglia C Giangreco M Verdoni L. Rheumatology Study Group of the Italian Pediatric Society. Defining Kawasaki disease and pediatric inflammatory multisystem syndrome-temporally associated to SARS-CoV-2 infection during SARS-CoV-2 epidemic in Italy: results from a national, multicenter survey. Pediatr Rheumatol Online J. (2021) 19:29. 10.1186/s12969-021-00511-7 33726806 PMC7962084

[9] Toubiana J Poirault C Corsia A Bajolle F Fourgeaud J Angoulvant F. Kawasaki-like multisystem inflammatory syndrome in children during the covid-19 pandemic in Paris, France: prospective observational study. BMJ. (2020) 369:m2094. 10.1136/bmj.m2094 32493739 PMC7500538

[10] Morris SB Schwartz NG Patel P Abbo L Beauchamps L Balan S. case series of multisystem inflammatory syndrome in adults associated with SARS-CoV-2 infection - United Kingdom and United States, March-August 2020. MMWR Morb Mortal Wkly Rep. (2020) 69:1450–6. 10.15585/mmwr.mm6940e1 33031361 PMC7561225

[11] Farooqi KM Chan A Weller RJ Mi J Jiang P Abrahams E. Columbia University Interdisciplinary MIS-C Follow-up Program and the CUIMC Pediatric/Adult Congenital Heart Research Collaborative. Longitudinal Outcomes for Multisystem Inflammatory Syndrome in Children. Pediatrics. (2021) 148:e2021051155. 10.1542/peds.2021-054181 34266903

[12] Feldstein LR Tenforde MW Friedman KG Newhams M Rose EB Dapul H. Overcoming COVID-19 Investigators. Characteristics and Outcomes of US Children and Adolescents With Multisystem Inflammatory Syndrome in Children (MIS-C) Compared With Severe Acute COVID-19. JAMA. (2021) 325:1074–87. 33625505 10.1001/jama.2021.2091 PMC7905703

[13] Information for Healthcare Providers about Multisystem Inflammatory Syndrome in Children (MIS-C) | CDC. Available at: https://www.cdc.gov/mis-c/hcp/ (accessed January 4, 2021).

[14] Penner J Abdel-Mannan O Grant K Maillard S Kucera F Hassell J. 6-month multidisciplinary follow-up and outcomes of patients with paediatric inflammatory multisystem syndrome (PIMS-TS) at a UK tertiary paediatric hospital: a retrospective cohort study. Lancet Child Adolesc Health. (2021) 5:473–82. 10.1016/S2352-4642(21)00138-3 34043958

[15] Choi NH Fremed M Starc T Weller R Cheung E Ferris A.. Pediatrics. (2020) 146:e2020009738. 10.1542/peds.2020-009738 33184170

[16] Lin JE Asfour A Sewell TB Hooe B Pryce P Earley C. Neurological issues in children with COVID-19. Neurosci Lett. (2021) 743:135567. 10.1016/j.neulet.2020.135567 33352286 PMC7831718

[17] Regan W O'Byrne L Stewart K Miller O Pushparajah K Theocharis P. Electrocardiographic changes in children with multisystem inflammation associated with COVID-19: associated with coronavirus disease 2019. J Pediatr. (2021) 234:27–32.e2. 10.1016/j.jpeds.2020.12.033 33358846 PMC7836928

[18] Capone CA Misra N Ganigara M Epstein S Rajan S Acharya SS. Six Month Follow-up of Patients With Multi-System Inflammatory Syndrome in Children. Pediatrics. (2021) 148:e2021050973. 10.1542/peds.2021-050973 34326176

[19] Barris DM Keelan J Ahluwalia N Jhaveri S Cohen J Stern K. Midterm outcomes and cardiac magnetic resonance imaging following multisystem inflammatory syndrome in children. J Pediatr. (2022) 241:237–41.e1. 10.1016/j.jpeds.2021.10.009 34687695

[20] Matsubara D Kauffman HL Wang Y Calderon-Anyosa R Nadaraj S Elias MD. Echocardiographic findings in pediatric multisystem inflammatory syndrome associated with COVID-19 in the United States. J Am Coll Cardiol. (2020) 76:1947–61. 10.1016/j.jacc.2020.08.056 32890666 PMC7467656

[21] Ahmed S Strait K RayChaudhuri N Gendi SM. Global longitudinal strain reduction in the absence of clinical cardiac symptoms in multisystem inflammatory syndrome in children associated with COVID-19: a case series. Pediatr Cardiol. (2022) 43:233–7. 10.1007/s00246-021-02712-z 34554265 PMC8459134

[22] Basu S Kim EJ Sharron MP Austin A Pollack MM Harahsheh AS. Strain echocardiography and myocardial dysfunction in critically Ill children with multisystem inflammatory syndrome unrecognized by conventional echocardiography: a retrospective cohort analysis. Pediatr Crit Care Med. (2022) 23:e145–52. 10.1097/PCC.0000000000002850 34636357 PMC8887681

[23] Kobayashi R Dionne A Ferraro A Harrild D Newburger J VanderPluym C. Detailed assessment of left ventricular function in multisystem inflammatory syndrome in children, using strain analysis. CJC Open. (2021) 3:880–7. 10.1016/j.cjco.2021.02.012 33649742 PMC7905387

[24] Ahmed M Advani S Moreira A Zoretic S Martinez J Chorath K. Multisystem inflammatory syndrome in children: a systematic review. EClinicalMedicine. (2020) 26:100527. 10.1016/j.eclinm.2020.100527 32923992 PMC7473262

[25] Haslak F Barut K Durak C Aliyeva A Yildiz M Guliyeva V. Clinical features and outcomes of 76 patients with COVID-19-related multi-system inflammatory syndrome in children. Clin Rheumatol. (2021) 40:4167–78. 10.1007/s10067-021-05780-x 34089099 PMC8178032

[26] Emeksiz S Özcan S Perk O Uyar E Çelikel Acar B Kibar Gül AE. Therapeutic plasma exchange: A potential management strategy for critically ill MIS-C patients in the pediatric intensive care unit. Transfus Apher Sci. (2021) 60:103119. 10.1016/j.transci.2021.103119 33836934

[27] Miller J Cantor A Zachariah P Ahn D Martinez M Margolis KG. Gastrointestinal symptoms as a major presentation component of a novel multisystem inflammatory syndrome in children that is related to coronavirus disease 2019: a single center experience of 44 cases. Gastroenterology. (2020) 159:1571–4.e2. 10.1053/j.gastro.2020.05.079 32505742 PMC7270806

[28] Lo Vecchio A Garazzino S Smarrazzo A Venturini E Poeta M Berlese P. Italian SITIP-SIP Paediatric SARS-CoV-2 Infection Study Group. Factors associated with severe gastrointestinal diagnoses in children with SARS-CoV-2 infection or multisystem inflammatory syndrome. JAMA Netw Open. (2021) 4:e2139974. 34928354 10.1001/jamanetworkopen.2021.39974 PMC8689385

[29] Lee MS Liu YC Tsai CC Hsu JH Wu JR. Similarities and differences between COVID-19-related multisystem inflammatory syndrome in children and Kawasaki Disease. Front Pediatr. (2021) 9:640118. 10.3389/fped.2021.640118 34222140 PMC8249705

[30] Syrimi E Fennell E Richter A Vrljicak P Stark R Ott S. The immune landscape of SARS-CoV-2-associated Multisystem Inflammatory Syndrome in Children (MIS-C) from acute disease to recovery. iScience. (2021) 24:103215. 10.1016/j.isci.2021.103215 34632327 PMC8487319

[31] Suksatan W Chupradit S Yumashev AV Ravali S Shalaby MN. Immunotherapy of multisystem inflammatory syndrome in children (MIS-C) following COVID-19 through mesenchymal stem cells. Int Immunopharmacol. (2021) 101:108217. 10.1016/j.intimp.2021.108217 34627083 PMC8487784

[32] Henderson LA Canna SW Friedman KG Gorelik M Lapidus SK Bassiri H. American College of Rheumatology Clinical Guidance for Multisystem Inflammatory Syndrome in Children Associated With SARS-CoV-2 and Hyperinflammation in Pediatric COVID-19: Version 2. Arthritis Rheumatol. (2021) 73:e13–29. 10.1002/art.41616 33277976 PMC8559788

[33] Newburger JW Takahashi M Gerber MA Gewitz MH Tani LY Burns JC. Diagnosis, treatment, and long-term management of Kawasaki disease: a statement for health professionals from the Committee on Rheumatic Fever, Endocarditis and Kawasaki Disease, Council on Cardiovascular Disease in the Young, American Heart Association. Circulation. (2004) 110:2747–71. 10.1161/01.CIR.0000145143.19711.78 15505111

[34] Venkatesha GA Srinivas N Mohamedali S Chandrasekar S. Sudden cardiac death in a young boy with multisystemic inflammatory syndrome in children (MISC). BMJ Case Rep. (2021) 14:e242635. 10.1136/bcr-2021-242635 34413034 PMC8378390

[35] McCormick DW Richardson LC Young PR Viens LJ Gould CV Kimball A. Deaths in children and adolescents associated with COVID-19 and MIS-C in the United States. Pediatrics. (2021) 148:e2021052273. 10.1542/peds.2021-052273 34385349 PMC9837742

[36] Abrams JY Oster ME Godfred-Cato SE Bryant B Datta SD Campbell AP. Factors linked to severe outcomes in multisystem inflammatory syndrome in children (MIS-C) in the USA: a retrospective surveillance study. Lancet Child Adolesc Health. (2021) 5:323–31. 10.1016/S2352-4642(21)00050-X 33711293 PMC7943393

[37] Zambrano LD Newhams MM Olson SM Halasa NB Price AM Boom JA. Effectiveness of BNT162b2 (Pfizer-BioNTech) mRNA Vaccination Against Multisystem Inflammatory Syndrome in Children Among Persons Aged 12-18 Years - United States, July-December 2021. MMWR Morb Mortal Wkly Rep. (2022) 71:52–8. 35025852 10.15585/mmwr.mm7102e1 PMC8757620

[38] Levy M Recher M Hubert H Javouhey E Fléchelles O Leteurtre S. Multisystem inflammatory syndrome in children by COVID-19 vaccination status of adolescents in France. JAMA. (2022) 327:281–3. 10.1001/jama.2021.23262 34928295 PMC8689418

[39] Davies P du Pré P Lillie J Kanthimathinathan HK. One-year outcomes of critical care patients post-COVID-19 multisystem inflammatory syndrome in children. JAMA Pediatr. (2021) 175:1281–3. 10.1001/jamapediatrics.2021.2993 34459875 PMC8406209

[40] Matsubara D Chang J Kauffman HL Wang Y Nadaraj S Patel C. Longitudinal assessment of cardiac outcomes of multisystem inflammatory syndrome in children associated with COVID-19 infections. J Am Heart Assoc. (2022) 11:e023251. 10.1161/JAHA.121.023251 35043684 PMC9238494

[41] Blondiaux E Parisot P Redheuil A Tzaroukian L Levy Y Sileo C. Cardiac MRI in children with multisystem inflammatory syndrome associated with COVID-19. Radiology. (2020) 297:E283–8. 10.1148/radiol.2020202288 32515676 PMC7294821

[42] Bartoszek M Małek ŁA Barczuk-Falecka M Brzewski M. Cardiac magnetic resonance follow-up of children after pediatric inflammatory multisystem syndrome temporally associated with SARS-CoV-2 with initial cardiac involvement. J Magn Reson Imaging. (2022) 55:883–91. 10.1002/jmri.27870 34327751 PMC8426796

[43] Sirico D Basso A Reffo E Cavaliere A Castaldi B Sabatino J. Early echocardiographic and cardiac mri findings in multisystem inflammatory syndrome in children. J Clin Med. (2021) 10:3360. 10.3390/jcm10153360 34362141 PMC8348478

[44] Prieto LM Toral B LLorente A Coca D Blázquez-Gamero D. Cardiovascular magnetic resonance imaging in children with pediatric inflammatory multisystem syndrome temporally associated with SARS-CoV-2 and heart dysfunction. Clin Microbiol Infect. (2021) 27:648–50. 10.1016/j.cmi.2020.10.005 33049415 PMC7547828

[45] Tannoury TE Bulbul ZR Bitar FF. Cardiac manifestations and short-term outcomes of multisystem inflammatory syndrome in Middle Eastern children during the COVID-19 pandemic: a case series. Cardiol Young. (2022) 32:165–8. 10.1017/S1047951121002614 34134808 PMC8280460

[46] Webster G Patel AB Carr MR Rigsby CK Rychlik K Rowley AH. Cardiovascular magnetic resonance imaging in children after recovery from symptomatic COVID-19 or MIS-C: a prospective study. J Cardiovasc Magn Reson. (2021) 23:86. 10.1186/s12968-021-00786-5 34193197 PMC8245157

[47] Hékimian G Kerneis M Zeitouni M Cohen-Aubart F Chommeloux J Bréchot N. Coronavirus disease 2019 acute myocarditis and multisystem inflammatory syndrome in adult intensive and cardiac care units. Chest. (2021) 159:657–62. 10.1016/j.chest.2020.08.2099 32910974 PMC7476896

[48] Chan KS Mangione-Smith R Burwinkle TM Rosen M Varni JW. The PedsQL: reliability and validity of the short-form generic core scales and Asthma Module. Med Care. (2005) 43:256–65. 10.1097/00005650-200503000-00008 15725982

[49] Paediatric Index of Emotional Distress - GL Assessment. Available at: https://www.gl-assessment.co.uk/assessments/products/paediatric-index-of-emotional-distress/ (accessed November 16, 2021).

[50] Maron BJ Zipes DP Kovacs RJ American American Heart Association Electrocardiography and Arrhythmias Committee of Council on Clinical Cardiology Council Council on Cardiovascular Disease in Young Council Council on Cardiovascular and Stroke Nursing . Eligibility and Disqualification Recommendations for Competitive Athletes With Cardiovascular Abnormalities: Preamble, Principles, and General Considerations: A Scientific Statement From the American Heart Association and American College of Cardiology. Circulation. (2015) 132:e256–61. 10.1161/CIR.0000000000000236 26621642

[51] COVID-19, Interim Guidance: Return to Sports. Available at: https://services.aap.org/en/pages/2019-novel-coronavirus-covid-19-infections/clinical-guidance/covid-19-interim-guidance-return-to-sports/ (accessed November 28, 2020)

[52] Chowdhury D Fremed MA Dean P Glickstein JS Robinson J Rellosa N. Return to activity after SARS-CoV-2 infection: cardiac clearance for children and adolescents. Sports Health. (2021) 24:19417381211039746. 10.1177/19417381211039746 34427496 PMC9214892

[53] Elliott N Martin R Heron N Elliott J Grimstead D Biswas A. Infographic. Graduated return to play guidance following COVID-19 infection. Br J Sports Med. (2020) 54:1174–5. 10.1136/bjsports-2020-102637 32571796 PMC7371566

[54] COVID-19 Interim Guidance: Return to Sports. Available at: https://services.aap.org/en/pages/2019-novel-coronavirus-covid-19-infections/clinical-guidance/covid-19-interim-guidance-return-to-sports/ (accessed November 25, 2020).

[55] Henderson LA Canna SW Friedman KG Gorelik M Lapidus SK Bassiri H. American College of Rheumatology Clinical Guidance for Multisystem Inflammatory Syndrome in Children Associated With SARS-CoV-2 and Hyperinflammation in Pediatric COVID-19: Version 3. Arthritis Rheumatol. (2022). 10.1002/art.42062. [Epub ahead of print]. 35118829 PMC9011620

[56] Truong DT Trachtenberg FL Pearson GD Dionne A Elias MD Friedman K. The NHLBI Study on Long-terM OUtcomes after the Multisystem Inflammatory Syndrome In Children (MUSIC): design and objectives. Am Heart J. (2022) 243:43–53. 10.1016/j.ahj.2021.08.003 34418362 PMC8710361

[57] RECOVER. Available at: https://recovercovid.org/ (accessed November 15, 2021).

